# Supplementary material for: From fangs to antidotes: A scoping review on snakebite burden, species, and antivenoms in the Eastern Mediterranean Region
Source: PLoS Negl Trop Dis. 2024 Jul 31;18(7):e0012200. doi: 10.1371/journal.pntd.0012200 (PMC11335162; doi:10.1371/journal.pntd.0012200)
Supplement: S2 Supplement — (DOCX) [file pntd.0012200.s002.docx]

**Supplement II: Search strategies**

| *Database* | PubMed |
| --- | --- |
| *Date of search* | 13-02-2023 |
| *Search query* | ((snakebite* OR snake bite* OR snake-bite* OR venomous bite* OR snake envenom* OR ophidian envenom*) OR ("snake bites"[Mesh])) AND (("Eastern Mediterranean" OR Eastern-Mediterranean OR Middle East* OR Arab* OR North Africa* OR Levant* OR Maghreb OR "Horn of Africa" OR Afghanistan* OR Bahrain* OR Djibouti OR Egypt* OR Iran* OR Iraq* OR Jordan* OR Kuwait OR Leban* OR Libya* OR Morocco* OR Oman* OR Pakistan* OR Palestin* OR Qatar* OR Saudi OR Somali* OR Sudan* OR Syria* OR Tunisia* OR Emirat* OR Yemen*) OR ("Middle East"[Mesh]) OR ("Africa, Northern"[Mesh]) OR ("Arabs/statistics and numerical data"[Mesh]) OR ("Iran"[Mesh]) OR ("Pakistan"[Mesh]) OR ("Afghanistan"[Mesh]) OR ("Djibouti"[Mesh]) OR ("Somalia"[Mesh]) OR ("Egypt"[Mesh]) OR ("Libya"[Mesh]) OR ("Morocco"[Mesh]) OR ("Tunisia"[Mesh]) OR ("Iraq"[Mesh]) OR ("Lebanon"[Mesh]) OR ("Syria"[Mesh]) OR ("Bahrain"[Mesh]) OR ("Kuwait"[Mesh]) OR ("Saudi Arabia"[Mesh]) OR ("Sudan"[Mesh]) OR ("United Arab Emirates"[Mesh]) OR ("Yemen"[Mesh]) OR ("Oman"[Mesh]) OR ("Jordan"[Mesh])) Filters: from 2000 - 2023 |
| *Number of hits* | 600 |

| *Database* | Web of Science |
| --- | --- |
| *Date of search* | 14-02-2023 |
| *Search query* | (ALL=(snakebite* OR snake bite* OR snake-bite* OR venomous bite* OR snake envenom* OR ophidian envenom*)) AND ALL=("Eastern Mediterranean" OR Eastern-Mediterranean OR Middle East* OR Arab* OR North Africa* OR Levant* OR Maghreb OR "Horn of Africa" OR Afghanistan* OR Bahrain* OR Djibouti OR Egypt* OR Iran* OR Iraq* OR Jordan* OR Kuwait OR Leban* OR Libya* OR Morocco* OR Oman* OR Pakistan* OR Palestin* OR Qatar* OR Saudi OR Somali* OR Sudan* OR Syria* OR Tunisia* OR Emirat* OR Yemen* ) |
| *Number of hits* | 612 |

| *Database* | CINAHL |
| --- | --- |
| *Date of search* | 14-02-2023 |
| *Search query* | "( (MH "Snake Bites") OR (MH "Snake Venoms") OR "snakebite* OR snake bite* OR snake-bite* OR venomous bite* OR snake envenom* OR ophidian envenom*" ) AND ( (MH "Egypt") OR (MH "Africa, Northern+") OR (MH "Middle East+") OR ""Eastern Mediterranean" OR Eastern-Mediterranean OR Middle East* OR Arab* OR North Africa* OR Levant* OR Maghreb OR "Horn of Africa" OR Afghanistan* OR Bahrain* OR Djibouti OR Egypt* OR Iran* OR Iraq* OR Jordan* OR Kuwait OR Leban* OR Libya* OR Morocco* OR Oman* OR Pakistan* OR Palestin* OR Qatar* OR Saudi OR Somali* OR Sudan* OR Syria* OR Tunisia* OR Emirat* OR Yemen*" OR (MH "Arabs") OR (MH "Yemen") OR (MH "Oman") OR (MH "Tunisia") OR (MH "Sudan") OR (MH "Qatar") OR (MH "Pakistan") OR (MH "Morocco") OR (MH "Libya") OR (MH "Kuwait") OR (MH "Jordan") OR (MH "Djibouti") OR (MH "Bahrain") OR (MH "Syria") OR (MH "Saudi Arabia") OR (MH "Afghanistan") OR (MH "Iraq") OR (MH "Iran") OR (MH "United Arab Emirates") ) Published Date: 20000101-20221231 AND Apply equivalent subjects on 2023-02-14 09:03 AM" |
| *Number of hits* | 30 |

| *Database* | IMEMR |
| --- | --- |
| *Date of search* | 06-04-2023 |
| *Search query* | tw:(snakebite* OR snake bite* OR snake-bite* OR venomous bite* OR snake envenom* OR ophidian envenom*) AND ( db:("IMEMR")) AND (year_cluster:[2000 TO 2023]) |
| *Number of hits* | 69 |
