# Supplementary material for: From fangs to antidotes: A scoping review on snakebite burden, species, and antivenoms in the Eastern Mediterranean Region
Source: PLoS Negl Trop Dis. 2024 Jul 31;18(7):e0012200. doi: 10.1371/journal.pntd.0012200 (PMC11335162; doi:10.1371/journal.pntd.0012200)
Supplement: S4 Supplement — (PDF) [file pntd.0012200.s004.pdf]

Supplement IV: Offending species by country

	Afghanistan	Djibouti	Egypt	Emirates	Iran	Iraq	Jordan	Lebanon	Libya	Morocco	Oman	Pakistan	Palestine	Qatar	Saudi Arabia	Sudan	Tunisia
Atractaspis engaddensis							x										
Atractaspis microlepidata																x	
Bitis arietans		x								x					x		
Bungarus caeruleus												x					
Cerastes cerastes									x	x				x	x		x
Cerastes gasperettii															x		
Cerastes vipera				x*													
Daboia mauritanica										x							
Daboia palaestinae							x	x									
Daboia russelii												x					
Echis carinatus	x				x							x				x*	
Echis coloratus							x				x		x		x		
Echis leucogaster										x							
Echis omanensis											x						
Echis pyramidum		x															
Hemorhois hypocrepis										x							
Hydrophis platurus												x					
Macrovipera Lebetina					x		x										x
Malpolon moilensis															x		
Montivipera bornmuelleri								x									
Montivipera latifii					x												
Naja haje			x							x							x
Naja oxiana	x																
Natrix maura										x							
Pseudocerastes fieldi							x										
Pseudocerastes persicus	x				x												
Vipera berus															x*		
Vipera latastei										x							x
Walterinnesia aegyptia							x								x		
Walterinnesia morgani						x											

TABLE 1 – SPECIES OF OFFENDING SNAKES BY COUNTRY AS PER INCLUDED RECORDS.

Footnote: The mark (*) denotes that the species did not accord WHO Snakebite Information and Data Platform and the herpetology guides consulted.
