# Supplementary material for: From fangs to antidotes: A scoping review on snakebite burden, species, and antivenoms in the Eastern Mediterranean Region
Source: PLoS Negl Trop Dis. 2024 Jul 31;18(7):e0012200. doi: 10.1371/journal.pntd.0012200 (PMC11335162; doi:10.1371/journal.pntd.0012200)
Supplement: Attachment — Submitted filename: Response to Reviewers.docx [file pntd.0012200.s005.docx]

Dear Editors,

We are sincerely grateful for the feedback on our manuscript entitled: “From fangs to antidotes: a scoping review on snakebite burden, species, and antivenoms in the Eastern Mediterranean Region” [PNTD-D-23-01336] considered for publication in PLOS Neglected Tropical Diseases. We greatly appreciate the reviewers’ comments that helped us to strengthen the manuscript.

In response to reviewers, we have reworked highlighted sections in the marked copy. Additionally, we have detailed our responses to the reviewers' comments below, with manuscript page numbers provided in parentheses. We trust that we have addressed key points and look forward to your feedback.

Finally, we would like add Dr. David J Williams as a co-author of this manuscript. In our previous submission, we acknowledged Dr. David’s inputs in study design, interpretation of findings, and drafting the manuscript. Since he has continued to support the study throughout this stage, we believe his inclusion as a co-author is merited.

Best regards,

Study authors

**Reviewers’ comments**

**Reviewer 1.1. Define Eastern Mediterranean countries; this can be in the form of a map.**

Response 1.1. Thank you so much for your feedback. We have added: “Stretching across large parts of North Africa and the Middle East all the way to Pakistan and Afghanistan, this region is home to nearly 767 million people [21], and encompasses 22 countries and territories [22] (shown in Figure 1)” (page 2). The included map depicts EMR countries and territories, as suggested.

**Reviewer 1.2. In the Supplement IV: Offending Species by country, the authors did not include Atractaspis, there are cases attributed to this snake in Jordan and Saudi Arabia. Also cases caused by Pseudocerastes fieldi were recorded from Jordan.**

Response 1.2. Thank you. The table in Supplement IV included only snake species that were identified in more than one publication, as formerly specified in page 13 of the manuscript. We made this decision because 15 species were reported only once each. As a result, the table became too wide when plotting 30 species half of which appearing only once. Eventually, we decided to include only species recorded more than once to enhance readability. We realize now that our previous decision might result in confusion and eliminate potentially important information. We have thus updated Supplement IV to include all species. The table now shows *Atractaspis* and *Pseudocerastes* species, among others, as you have noted.

**Reviewer 1.3. Citations of some references should be checked such as: Amr ZS, Abu Baker MA, Warrell DA. Terrestrial venomous snakes and snakebites in the 582 Arab countries of the Middle East. Toxicon Off J Int Soc Toxinology. 2020 Apr 15;177:1–15.**

Response 1.3. We appreciate your feedback. We have corrected this citation, and have checked all the references in the manuscript.

**Reviewer 1.4. Some important references on Jordan and Qatar are missing: Saadeh, A.M., 2001. Case report: acute myocardial infarction complicating a viper bite. Am. J. Trop. Med. Hyg. 64, 280–282. https://doi.org/10.4269/ajtmh.2001.64.280 for Jordan; Elmoheen, A., Salem, W. A., Haddad, M., Bashir, K., Thomas, S. H. 2020. Experience of Snakebite Envenomation by a Desert Viper in Qatar. Journal of Toxicology. https://doi.org/10.1155/2020/8810741**

Response 1.4. Thanks. The Jordanian paper by Saadeh was included in our review; please refer to Supplement III – Row #38. The paper on Qatar by Elmoheen et al. is a literature review that does not contain information on confirmed snakebite human victims. Therefore, it was not included as per our exclusion criterion #1.

**Reviewer 1.5. In the Supplement IV: it lists 15 species of snakes, while the authors stated that 30 species were Identified indigenous snakes.**

Response 1.5. Thank you. As we have explained in Response 1.2., the table in Supplement IV listed only species that were identified in more than one publication, as formerly specified in page 13 of the manuscript. We have updated Supplement IV to include all 30 species, and indicated so in the main body of the manuscript (p. 15).

**Reviewer 1.6. The time specified between 200-2023 caused some discrepancy in the other species that are considered venomous such as Naja sp. in both the Middle East and North Africa. At least these species should be mentioned.**

Response 1.6. We acknowledge your comment. We clarified in our Methods: “Second pillar was snake species i.e., offending species and snake identification methods. For this, one reviewer cross-referenced offending species from older publications against recent taxonomic revisions to ensure consistency in species identification” (p. 7). As for *Naja* snakes in our review, they were identified in publications from 2014-2021; thus, we believe the long timespan did not create a discrepancy for these particular species.

**Reviewer 1.7. First, I thank the authors for their efforts to outline this review, it is very important for all health officials of countries in the study area. However, the presentation of the results should be improved to facilitate the reader to better understand a sequential, ordered and graphical results. Graphs, maps and tables that can be extracted from the supplementary data should be incorporated to enhance the final format.**

Response 1.7. Thank you very much for your valuable comments. We revised and proofread the entire manuscript based on your comments, expanded the table in Supplement IV, included a map depicting EMR countries (p. 3), corroborated information on antivenoms’ specificity with WHO platform (p. 18), and elaborated on our discussion (p. 21-3). We hope that these will improve the readability and presentation of this paper.

**Reviewer 2.1. The manuscript "From fangs to antidotes: a scoping review on snakebite burden, species, and antivenoms in the Eastern Mediterranean Region is well written and reports on a region that has been neglected in regard to reports on snakebite.**

Response 2.1. We appreciate your review of the manuscript and very valuable feedback. We thank you for the time you have taken to help us improve it.

**Reviewer 2.2. At least one other figure should be added to help readers visualise the results, as there are few figures in this manuscript. A figure depicting the region studied on a map (all countries evaluated) would add to the manuscript, or could even be an additional panel in Figure 2.**

Response 2.2. Thank you for raising this point. We have added a map depicting EMR countries (p. 3).

**Reviewer 2.3. Additionally, maybe incorporation of information from venomous snake field guides in this region, it would be good to know what is being reported in the scientific literature vs information from herpetology guidebooks.**

Response 2.3. Thank you for your suggestion. We compared and found that the species findings from our review were mostly consistent with both herpetology and toxicology guides on the Region and WHO’s Snakebite Information and Data Platform except for three snakes. We made relevant additions to the methods (p. 7), results (p. 15) and discussion (p. 22).

**Reviewer 2.4. Lastly, this manuscript might be better considered a systematic review and not a primarily research article?**

Response 2.4. Thank you for your comment. We followed PLOS NTDs’ guidelines and publications which dedicate that a systematic/scoping review be submitted as a ‘Research Article.’
